# Ontogenetic Characterization of the Intestinal Microbiota of Channel Catfish through 16S rRNA Gene Sequencing Reveals Insights on Temporal Shifts and the Influence of Environmental Microbes

**DOI:** 10.1371/journal.pone.0166379

**Published:** 2016-11-15

**Authors:** Jacob W. Bledsoe, Brian C. Peterson, Kelly S. Swanson, Brian C. Small

**Affiliations:** 1 Center for Fisheries, Aquaculture, and Aquatic Sciences, Department of Animal Science, Southern Illinois University, Carbondale, IL, United States of America; 2 Warmwater Aquaculture Research Unit, USDA-ARS, Stoneville, MS, 38776, United States of America; 3 Department of Animal Sciences and Division of Nutritional Sciences, University of Illinois-Urbana Champaign, Urbana, IL, United States of America; 4 Aquaculture Research Institute, Department of Fish and Wildlife Sciences, University of Idaho, Hagerman, ID, United States of America; University of North Carolina at Chapel Hill, UNITED STATES

## Abstract

Aquaculture recently overtook capture fisheries as the largest producer of food fish, but to continue increasing fish production the industry is in search of better methods of improving fish health and growth. Pre- and probiotic supplementation has gained attention as a means of solving these issues, however, for such approaches to be successful, we must first gain a more holistic understanding of the factors influencing the microbial communities present in the intestines of fish. In this study, we characterize the bacterial communities associated with the digestive tract of a highly valuable U.S. aquaculture species, channel catfish *Ictalurus punctatus*, over the first 193 days of life to evaluate temporal changes that may occur throughout ontogenetic development of the host. Intestinal microbiota were surveyed with high-throughput DNA sequencing of 16S rRNA V4 gene amplicons derived from fish at 3, 65, 125, and 193 days post hatch (dph), while also characterizing the environmental microbes derived from the water supply and the administered diets. Microbial communities inhabiting the intestines of catfish early in life were dynamic, with significant shifts occurring up to 125 dph when the microbiota somewhat stabilized, as shifts were less apparent between 125 to 193 dph. Bacterial phyla present in the gut of catfish throughout ontogeny include Bacteroidetes, Firmicutes, Fusobacteria, and Proteobacteria; with the species *Cetobacterium somerae* and *Plesiomonas shigelloides* showing the highest abundance in the catfish microbiota after 3 dph. Comparisons of the gut microbiota to the environmental microbes reveals that the fish gut is maintained as a niche habitat, separate from the overall microbial communities present in diets and water-supply. Although, there is also evidence that the environmental microbiota serves as an inoculum to the fish gut. Our results have implications for future research related to channel catfish biology and culture, and increase our understanding of ontogenetic effects on the microbiota of teleost fish.

## Introduction

Aquaculture is currently the fastest growing food production sector globally, and growth is expected to continue as the industry attempts to meet the increasing demand of protein for human consumption [1]. To meet this growing demand, aquaculture production of finfish has intensified; however, this increase in the level of production has brought about new challenges in managing fish health and nutrition. The investigation of pre- and probiotics to overcome these issues has gained substantial research attention in aquaculture with some promising results; yet, studies typically only characterize effects at the level of the host’s systemic physiology (i.e. growth, immunity), ignoring the overall impacts at the microbial level [2]. In addition, results from probiotic studies are confounded by the diversity of species and culture techniques (e.g. feeding, environment, management strategies) utilized in the industry, often leading to inconclusive results [3]. This suggests that we must first gain a more holistic understanding of the factors that intrinsically influence the ecological composition of the intestinal microbiota of fish before implementing such strategies.

Research aimed at studying the microbiota in teleost fishes has greatly lagged behind that conducted on mammals; however, recently many studies have arisen including much comparative research conducted on zebrafish *Danio rerio* [4–6] as well as studies on various aquaculture species [7–9]. Common themes in these studies suggest that environmental factors such as water temperature [10], salinity [11], diet composition [12, 13], feeding strategy [14, 15], the type of systems utilized to culture the fish [16], and environmental presence of chemical agents [17–19] all exert selective forces on the microbial ecology of the digestive tract of fishes. In addition, intrinsic factors such as host physiology (e.g. stress, starvation, behavior) [20–22] and host genotype [23, 24] have been shown to drive differences in the structure of the intestinal microbiota. Yet, less research has explored the influence that host ontogeny has on the intestinal microbiota of fish.

Ontogenetic changes in the microbiota of fish should be of great interest in aquaculture, as cultured fish typically experience high levels of unpredictable mortality at early life stages; a phenomena that is likely associated with negative interactions between the environmental microbiota and the microbiota associated with fish larvae [25]. In addition, interactions between the host and the intestinal microbiota have been shown to play an integral role in proper ontogenetic development in vertebrates [26], especially in relation to the immune system [27–29]. The influence of host ontogeny on the intestinal microbiota has been investigated in zebrafish, with results demonstrating that multiple shifts occur as the fish develop over time, which are particularly influenced by changes in dietary requirements associated with age [13, 30]. However, these results from a comparative model species may not have direct applications in aquaculture. To date, only a few studies have explored the intestinal microbiota of aquaculture species from early larval stages through later developmental stages using molecular techniques. Yet, these studies have often been focused on marine species such as anadromous coho salmon *Oncorhynchus kisutch* [31] or marine Atlantic cod *Gadus morhua* [32], with only one such study evaluating the temporal development of the gut microbiota of a freshwater aquaculture species [33].

Channel catfish *Ictalurus punctatus* represent an ideal species for studying the microbiota of cultured fish species, as the production of this freshwater fish accounts for approximately 65% of the U.S. aquaculture industry, with over 300 million pounds processed annually [34]. The species is also widely accepted as a model for the study of immune function in teleost fish [35], due to the large body of research that exists relating to the species’ genetics, physiology, and immunology, which could facilitate future evaluations of host-microbiome interactions. Despite this fact, only one study to date has used molecular approaches to explore the structural dynamics of the intestinal microbiota in channel catfish [36], with no evaluations of ontogenetic effects. This indicates a need for more research on the intestinal microbiota of channel catfish, as such research is likely to have broad impacts with both basic and applied implications [37]. In this study, we explore changes to the intestinal microbiota of channel catfish across developmental ontogeny by surveying the microbes associated with catfish, as well as the water supply and administered diets, at four distinct time points (3, 65, 125, and 193 days post hatch (dph)) using high-throughput 16S rRNA gene sequencing. Results have implications on the management of disease, nutrition, and probiotic use in the channel catfish aquaculture industry, as well as serve to further unravel the temporal variability and influence of host ontogeny and environment associated with the intestinal microbiota of teleost fish.

## Materials and Methods

### Ethics Statement

Samples used in this study were collected from aquacultured channel catfish humanely euthanized in a buffered solution of tricaine methanesulfonate (MS-222; Western Chemical Inc., Furndale, WA, USA). All animal protocols, including sample collection, animal handling and husbandry, were reviewed and approved by the Institutional Animal Care and Use Committee (IACUC) of the USDA-ARS Warmwater Aquaculture Research Unit (Protocol # 64-F-006-6803).

### Fish Husbandry

All fish husbandry was conducted at the USDA-ARS Warmwater Aquaculture Research Unit (WARU) (Stoneville, MS, USA). All fish used in this experiment were Delta Select strain channel catfish, a selectively crossed strain developed by the USDA-ARS through random mating of progeny obtained from eight hatcheries located in the Mississippi Delta, the epicenter of catfish aquaculture production within the United States. The genetic diversity encompassed within this strain is intended to be representative of the common genetics found across the majority of the U.S. channel catfish aquaculture industry. Eggs were collected from a single spawning event of one family of Delta Select channel catfish from an outdoor pond at the USDA-ARS WARU in early July, disinfected in a 100 ppm Povidone-iodine solution following industry standards [38], and brought indoors. After hatching, approximately 500 sac-fry larvae were randomly collected for inclusion in our study population and were placed into a separate indoor 76L flow-through tank, supplied with aerated well water (~26°C, pH ~ 8.5, dissolved oxygen > 5ppm), for the remainder of the experiment. As the fish grew, fish density was maintained within the tank by random culling of individuals when necessary. All fish were fed daily to apparent satiation using commercially available diets. Over the 193-day trial, three different diet formulations were administered in order to meet the dynamic dietary protein requirements dictated by the life history of channel catfish (Table 1). Diet formulations remained constant between sampling points, but changed immediately following each sampling period. Although diet formulations were consistent, diet pellet size was gradually increased to meet the increasing gape size of growing fish. This resulted in three pellet sizes for the Starter (fed from first feeding to 65 dph), two for the Extr 450 (fed from 65 to 125 dph), and one for the MiniPellet (fed from 125 to 193 dph), totaling in six unique dietary samples (Table 1). To evaluate any shifts in the intestinal microbiota of channel catfish across ontogenetic development, a single family of fish was repeatedly sampled at 3, 65, 125, and 193 dph. Samples were taken from full-sib individuals raised in a communal tank to reduce the potential influence of host-genetics and environment on the microbiota.

**Table 1:** Proximate analysis of diets fed to channel catfish throughout the study. Proximate analysis was conducted in duplicate for each sample, with averages displayed. Starter was administered from first feeding to 65 dph, Extr450 between 65 and 125 dph, and MiniPellet between 125 and 193 dph.

Diet	Pellet Size (mm)	Dry Matter (%)	Crude Protein (%)	Crude Fat (%)	Ash (%)
Starter^A^	0.6–1.4*	91.0	54.6	15.1	10.0
Extr 450^A^	1.4–1.7*	89.7	46.0	14.2	11.7
MiniPellet^B^	2.4	90.7	34.4	4.5	8.0

^A^ Rangen Inc., ID, US

^B^ Fishbelt Feeds Inc., MS, US.

^*^ Diet formulations fed at increasing pellet sizes over time.

### Fish Sampling and Isolation of Microbial DNA

At all time points, a total of ten fish were sampled and processed individually. At 3 dph, sac-fry larvae were euthanized with 200 mg L^-1^ of tricaine methanesulfonate (MS-222; Western Chemical Inc., Furndale, WA, USA) buffered with equal parts sodium bicarbonate, in water taken from the culture tank. Because sac-fry larvae were too small to isolate the intestinal tract, a DNA removal treatment was used to remove external microbes from euthanized larvae, so that a representative sample of the internal microbiota could be gained using whole larvae. The treatment consisted of submerging the euthanized larvae for thirty seconds in an alkaline DNA and DNase removal solution (DNase Displace Decontaminant, ThermoFisher Scientific, Wilmington, DE, USA) followed by a brief rinse with 70% ethanol. Immediately following the rinse, larvae were placed directly into sterile microcentrifuge tubes, immediately flash frozen, and stored at -80°C until further processing. Representative weights and lengths of sac-fry were obtained from ten separate sac-fry larvae at the time of sampling.

At all other time points, fish were large enough to enable the intestinal tract to be isolated using sterile procedures. At 65, 125, and 193 dph, fish were euthanized via submersion in 300 mg L^-1^ of tricaine methanesulfonate buffered with equal parts sodium bicarbonate in water taken from the culture tank, with fish remaining in the solution until ten-minutes following cessation of opercular movement [39]. Following euthanasia, length and weight were recorded and the ventral surface of the fish was washed with 70% molecular grade ethanol, before a sterile incision was made from the pelvic girdle to the cloacal pore. Using sterile procedures, the entire intestinal tract and its contents were removed just dorsal of the pylorus to directly anterior of the cloaca. Samples were placed individually in sterile tubes, flash frozen in liquid nitrogen, and then stored at -80°C until further processing. Disposable sterile scalpels were used individually on each fish, and all other dissection tools were rinsed with DNase Displace Decontaminant and 70% ethanol between samples.

In order to homogenize samples for DNA extraction, whole sac-fry and whole intestinal samples were ground individually to a homogenous dry powder using separate sterilized and autoclaved mortar and pestles, partially submerged in liquid nitrogen. This technique allowed both fecal and mucosal-epithelium associated microbes to be captured for DNA isolations using the PowerFecal® DNA Isolation Kit (MO BIO Laboratories Inc., Carlsbad, California, USA) following the manufacturer’s protocol. Isolated DNA was checked for quality and concentration using a NanoDrop 2000c (ThermoFisher Scientific, Wilmington, DE, USA) and each sample was run alongside a 1 kb + ladder (Invitrogen, Grand Island, NY, USA) on a 1% agarose gel to assess DNA degradation.

### Environmental Sample Collection and Processing

Samples were collected from all diets fed throughout the study and from the water source supplying the culture tank, in order to evaluate the potential influence of the environmental microbes on the intestinal microbiota. A single sample was taken from each of the six commercial diets that were fed to the fish throughout the trial, and were stored in sterile tubes at -20°C until further processed. Diet samples were homogenized and processed for DNA isolation using the same methods as described for the intestinal samples. Proximate analysis was also conducted on the diets, following AOAC protocols [40] (Table 1).

Samples were also collected from the inflowing water supplied to the fish culture tank, which originated from a consistent well-water source (Mississippi River Valley Alluvial Aquifer). Unfortunately, water samples taken at 3, 65, and 125 dph were contaminated during sample storage, therefore, the water sample collected at the last sampling point (193 dph) was used to represent the water microbiota over the duration of the study. From this representative water sample, two 250 mL aliquots were filtered through 0.45 μm sterile cellulose vacuum filters (MO BIO Laboratories Inc., Carlsbad, California, USA) to capture microbes associated with the water supplied to the fish culture tank. Filters were then removed and placed into sterile tubes for DNA extraction using the PowerWater® DNA isolation kit (MO BIO Laboratories Inc., Carlsbad, California, USA), following the manufacturer’s recommended protocol. Quality and concentration of isolated DNA was analyzed using the same methods previously described. In addition, a negative control water sample was prepared following the same protocol, using sterile molecular grade water to determine contamination from the DNA extraction process.

### 16S rRNA Library Preparation and Sequencing

Following extraction and quality checks, DNA samples were submitted to the Roy J. Carver Biotechnology Center (University of Illinois, Champaign, IL, USA) for amplicon library preparation and high-throughput sequencing. Briefly, the Fluidigm Access Array (Fluidigm, San Francisco, CA, USA), a microfluidic high-throughput multiplexed PCR system, was used to construct 16S rRNA V4 sequencing amplicons, using 505f and 806r PCR primers, Fluidigm specific spacer pads (CS1 and CS2), 10 nucleotide (nt) barcodes unique to each sample, and Illumina specific sequencing primers (i5 and i7) (S1 Fig). Barcoded amplicons were pooled for library construction using the TruSeq DNA Sample Prep kit (Illumina, San Diego, CA, USA), with library validation conducted using qPCR and an Agilent Bioanalyzer (Agilent, Santa Clara, CA, USA). High-throughput sequencing was conducted on the Illumina MiSeq platform (Illumina, San Diego, CA, U.S.A) using v3 chemistry to yield 315 nt paired-end (PE) reads.

### Bioinformatics

Paired-end reads were merged using PEAR v0.9.6 [41] with a minimum assembly length of 250 nt and a quality score threshold of 25 Phred. The FASTX-Toolkit (http://hannonlab.cshl.edu/fastx_toolkit/index.html) was then used to trim PCR primers from the PE reads. All further bioinformatics analyses were conducted using QIIME 1.9.1 [42]. Sequences were demultiplexed according to assigned 10 nt Fluidigm specific barcodes. Chimeric sequences were identified and removed using UCHIME [43], before clustering sequences into Operational Taxonomic Units (OTUs) and assigning taxonomy using open reference UCLUST [44] with PyNAST alignment [45] against GreenGenes v13.8 [46] at a 97% sequence identity. Singletons, or OTUs represented by only one sequence read, were removed from analysis. Clusters assigned to the order chloroplast or the family mitochondria were removed from analysis, as they represented reads derived from plant material found in the diets and eukaryotic and host mitochondrial DNA, respectively, and were not considered functional microbiota [47]. We further required OTUs to account for at least 0.0001% of the overall abundance, in order to reduce the incidence of spurious OTUs. Rarefaction analysis was then used to determine the even sampling depth at which the greatest number of gut samples could be retained, while still observing a leveling trend in the rarefaction curves. Based upon this analysis the rarefaction limit was set to 2,798 OTUs (S2 Fig), and all samples containing OTUs below this level were removed from further statistical analysis.

#### Data availability

Raw sequencing reads from all samples included in the analysis can be openly accessed on the NCBI database (http://www.ncbi.nlm.nih.gov/) under BioProject accession number PRJNA329560.

### Statistical Analysis

#### Alpha diversity

In QIIME, alpha diversity was calculated for each sample using three different indices: the number of observed OTUs, Chao1 species richness estimator, and distance-based whole-tree phylogenetic diversity. Statistical analysis of alpha diversity indices associated with fish was conducted across all gut samples in R by one-way ANOVA with a Tukey’s HSD post-hoc test. Assumptions of normality and homogeneity of variance were assessed using a Shapiro-Wilks test and Bartlett’s test, respectively, leading to a log transformation of Chao1 richness estimations.

#### Beta diversity

Beta diversity was determined through QIIME by calculating the unweighted UniFrac distance, an index which functions on the presence or absence of OTUs between samples, while considering the phylogenetic relatedness of the various microbiota. All statistical comparisons of beta diversity were conducted using Primer v7 (Primer-E Ltd, Plymouth, UK). To visually display patterns of beta-diversity, principle coordinate analysis (PCoA) plots were generated using UniFrac distances. In addition, group centroids were plotted using non-metric multidimensional scaling (nMDS) to visually observe the general trajectory of beta diversity across the time points (groups) sampled. A PERMANOVA statistical test was used to analyze beta diversity across time (fixed factor of fish age; four groups, 3DPH, 65DPH, 125DPH, and 193DPH). This test was employed because of the non-parametric skewed nature of microbial ecology surveys, which often excludes the use of traditional multivariate analysis such as MANOVA. PERMANOVA, a non-parametric permutation-based multivariate ANOVA, employs non-Euclidian distances, such as UniFrac, as a measure of multivariate data and makes no assumptions other than the null hypothesis that samples are completely interchangeable [48]. A one-way ordered analysis of similarity (ANOSIM) was also conducted on UniFrac distances to accompany the results of the PERMANOVA, as ANOSIM test statistics are more comparable across studies and give further information on how distinguishable groups are from one another by incorporating within- and between- group variability [49]. In addition, a PERMDISP test, a non-parametric multivariate equivalent to a traditional Levene’s test, was conducted under the null hypothesis of no differences of within group multivariate dispersion across sampling time points, to give insight on within- and between-group dispersion to enable more accurate interpretation of the PERMANOVA and ANOSIM results [50]. PERMANOVA, ANOSIM, and PERMDISP tests were conducted using 9,999 permutations of data, following the recommendation of Clarke and Gorley [51]. When statistical tests identified significant effects within the main test (P ≤ 0.05), pairwise tests were conducted to determine which specific time points attributed significantly to the detected differences.

#### Differential abundance of OTUs

To identify the bacterial taxa that were differentially abundant across catfish ontogeny and potentially driving the difference in beta diversity, similarity percentage analysis (SIMPER) was conducted using Primer v7. For this analysis, OTUs were required to be detected in three or more separate fish samples, to remove rare taxa, before conducting the SIMPER analysis on a Bray-Curtis similarity matrix assembled from a genus level OTU table. And because SIMPER does not explicitly employ statistical tests, the genus level OTU table was further analyzed by the least discriminant analysis (LDA) effect size (LEfSe) biomarker discovery module (available at http://huttenhower.sph.harvard.edu/galaxy/) to statistically test for differentially abundant OTUs across catfish ontogeny. The LEfSe analysis was conducted using the all-against-one model under default parameters (Kruskal-Wallis test P ≤ 0.05 and log 10 LDA threshold = 2.0), with fish age serving as the class, individual sample IDs as the subject, and no declared subclass.

#### Influence of environmental microbiome

Two-tailed Welch t-tests were performed on alpha diversity metrics between the fish samples and environmental samples at each time point. PCoA plots were generated based on unweighted UniFrac beta diversity distances for each time point including the fish samples, the two representative water-supply samples, and all diets unique to that time point. One-way ANOVAs were conducted on the unweighted UniFrac distances between fish samples and the replicate water-supply samples, as well as the diets unique to each time point.

## Results

**Fig 1:**
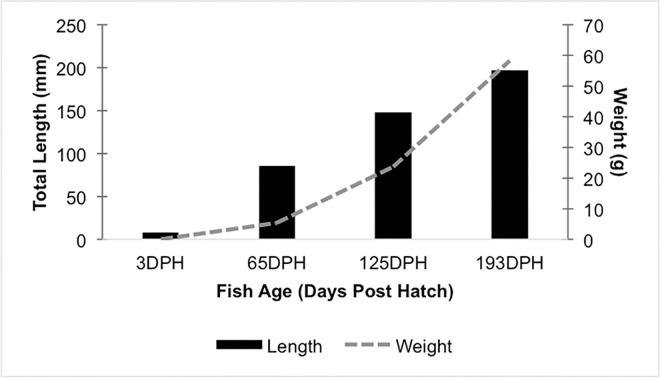
Weights and lengths of channel catfish sampled at 3, 65, 125, and 193 days post hatch (DPH). Mean fish weights (grams) plotted as a solid line and mean total length (mm) displayed as bars. Ten fish were measured at each time point.

All water quality parameters were maintained within an acceptable range for the duration of this study, and no fish health issues were detected in the study population throughout the experiment. Fish growth followed expectations, with weights increasing from (mean ± SD) 0.012 ± 0.005 g at 3 dph, to 58.15 ± 12.57 g by the end of the trial at 193 dph (Fig 1). After quality filtering and processing sequencing reads, the sample size of fish at each time point was reduced by removal of samples which fell below the rarefaction limit. This resulted in a final sample size of five fish at 3 dph, seven fish at 65 dph, four fish at 125 dph, and six fish at 193 dph. From those twenty-three fish samples, a total of 783,984 PE sequencing reads were clustered into 209 distinct OTUs after filtering out non-functional taxa and low abundance OTUs.

### Alpha Diversity

**Fig 2:**
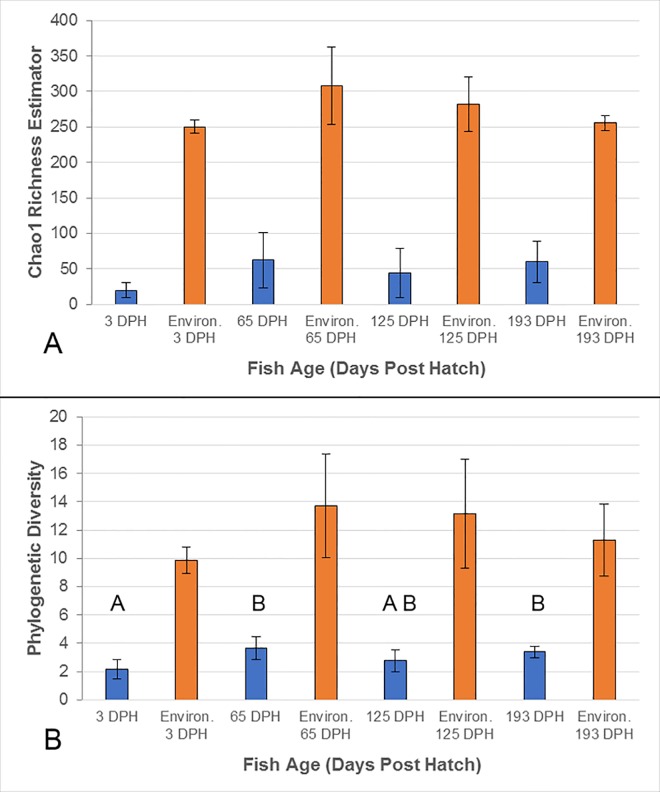
Alpha diversity of the intestinal microbiota of channel catfish and the combined environmental microbiota (administered diets and water-supply) at each sampling point (days post hatch, DPH). Bars represent the group means of fish samples (blue) and the environmental samples associated with fish at each time point (orange), with error bars displaying the SD. Alpha diversity is represented by Chao1 species richness estimator (A) and whole-tree phylogenetic diversity (B). An ANOVA followed by Tukey’s post-hoc test was conducted across all intestinal samples to detected differences in alpha diversity over time (P ≤ 0.05). No differences were detected for Chao 1 (A), while letters indicate significant differences detected in phylogenetic distance (B). Two-tailed Welch t-tests detected significant differences (P < 0.05) between the intestinal samples and the combined environmental samples at all time points, based upon either index.

The number of observed OTUs detected from the catfish intestines in this study was relative low overall, with a great deal of individual variation between cohorts, resulting in no significant differences across the four age groups (P = 0.198; S2 Fig). Similarly, no statistical differences were detected across fish age based on Chao1 species richness estimates (P = 0.112; Fig 2A). However, whole-tree phylogenetic diversity (PD) did show greater microbial diversity at 65 and 193 dph as compared to the two other age groups (P < 0.05, Fig 2B).

### Beta Diversity

**Fig 3:**
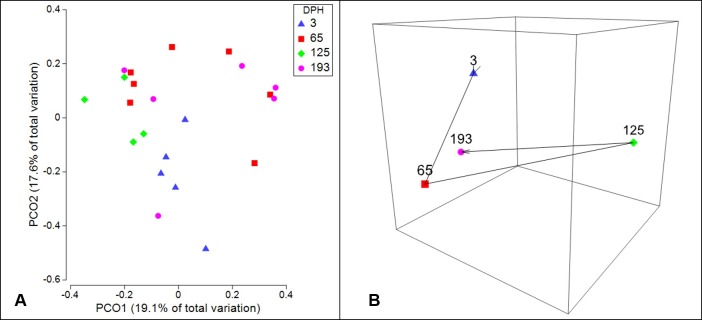
Visual presentations of the ontogeny of the intestinal microbiota of channel catfish using unweighted UniFrac beta diversity distances. (A) Principal coordinates analysis (PCoA) plots of unweighted UniFrac distances obtained from the intestinal microbiota of channel catfish at four distinct time points: 3, 65, 125, and 193 days post hatch (DPH). Points represent individual samples. (B) Non-metric multidemsional scaling (nMDS) plot showing the trajectory of the intestinal microbiota of channel catfish sampled over the first 193 dph. Individual points represent the group centroid (distance based multivariate group mean) of each time point sampled.

Visual representation of beta diversity using PCoA shows moderate grouping of individual catfish gut microbiota samples within time points, yet much overlap across time points is apparent as well (Fig 3A). Condensing samples from each time point down to multivariate group centroids based on unweighted UniFrac distance, reveals that the microbiota composition was especially divergent from the other time points at 125 dph, yet by 193 dph the group centroid was realigned with the earlier time points (Fig 3B). Further, statistical analysis of beta diversity across catfish ontogeny shows significant divergence of the microbial communities present in the gut across fish age, as both PERMANOVA and ANOSIM main tests indicate a significant difference (P ≤ 0.001; Table 2A). In addition, the non-significant results of the PERMDISP test (P ≥ 0.05; Table 2A) indicated that within group dispersions were homogenous, therefore the results of the PERMANOVA can be interpreted as true differences in multivariate location, or composition of microbial communities. Furthermore, the pairwise PERMANOVA results are in agreement that significant shifts in beta diversity occurred between each time point that catfish were sampled (P ≤ 0.05; Table 2B), with the exception of the period from 125 dph to 193 dph (P = 0.0705; Table 2B). The largest difference in microbial composition across fish age was detected between 65 dph and 125 dph, as this comparison showed the largest ANOSIM test statistic (R^O^ = 0.68, Table 2B), which serves as a measure of separation with an R statistic of 1 representing complete dissimilarity. Interestingly, no significant differences were detected when comparing the microbial communities of 193 dph catfish to any of the other three time points (Table 2B).

**Table 2:** Main (A) and pairwise test results (B) of non-parametric permutation-based multivariate statistical analysis of unweighted UniFrac beta diversity of the intestinal microbiota of channel catfish across age (days post hatch; DPH). All statistical tests were conducted using a fixed factor of fish age, across four groups: 3DPH, 65DPH, 125DPH, and 193DPH. Test statistics were calculated using up to 9,999 permutations, yet data structure dictated the number of possible permutations.

**A**				
**Main Test Across Fish Ages**				
	**Statistical Test**	**Test Statistic**	**P-value**	**Possible Permutations**
	**PERMDISP**	0.8411	0.6501	9999
	**PERMANOVA**	2.1266	*0.0004	9890
	**ANOSIM** (ordered)	0.3090	*0.0002	9999
**B**				
**Pairwise Tests Between Fish Ages**				
**Statistical test**	**Groups Compared**	**Test Statistic**	**P-value**	**Possible Permutations**
**PERMANOVA**	3DPH and 65DPH	1.569	*0.0169	792
	65DPH and 125DPH	1.819	*0.0037	330
	125DPH and 193DPH	1.339	0.0705	210
	3DPH and 125DPH	1.515	*0.0071	126
	3DPH and 193DPH	1.298	0.0741	460
	65DPH and 193DPH	1.242	0.1130	1709
**ANOSIM**	3DPH and 65DPH	0.386	*0.009	792
	65DPH and 125DPH	0.680	*0.003	330
	125DPH and 193DPH	0.190	0.138	210
	3DPH and 125DPH	0.400	*0.004	126
	3DPH and 193DPH	0.136	0.154	460
	65DPH and 193DPH	0.135	0.107	1709

*—Indicates a rejection of the null hypothesis of no differences among groups (P ≤ 0.05)

### Temporal Variability and Differential OTUs

**Fig 4:**
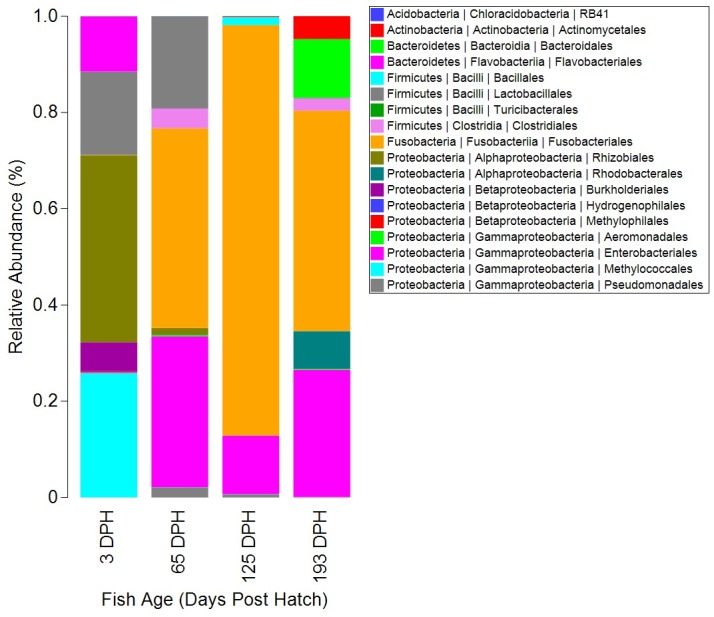
Relative abundance of bacterial orders detected in the intestinal microbiota of channel catfish sampled at 3, 65, 125, and 193 days post hatch (DPH). Bars represent the averages taken from all fish included in analysis at each time point. Legend indicates hierarchical taxonomy of OTUs at three taxonomic levels (phyla | class | order). Microbiota were filtered to remove chloroplast OTUs derived from plant material in the diets, and eukaryotic mitochondrial reads, as well as removing OTUs representing less than 0.0001% of the total abundance.

To further explore microbial differences through time, SIMPER analysis was used to evaluate within-group similarity and among-group dissimilarity, after removing taxa which were not detected in at least three separate gut samples. This analysis identified the bacterial taxa that were most typical of each age group, as well as those most discriminatory between groups (Table 3). Within-group similarity was lowest at 3 dph (9.72%), but increased at 65 dph (15.65%) and 125 dph (66.20%), before falling again at 193 dph (35.48%). In addition, SIMPER indicated that Proteobacteria from the genus *Bradyrhizobium* were most characteristic of the larval microbiome at 3 dph. *Streptococcus* from the phyla Firmicutes, as well as *Plesiomonas shigelloides* from the phyla Proteobacteria were identified as representative of the 65 dph catfish gut microbiota. At 125 dph, the only species of Fusobacteria detected in the catfish intestine, *Cetobacterium somerae*, dominated the gut microbiota with an average relative abundance of 83% (Fig 4). *C*. *somerae* was also found to be a characteristic OTU of the 193 dph catfish microbiota, in addition to *P*. *shigelloides*. Among-group dissimilarity analysis reveals the microbiota of 3 dph sac-fry larvae as the most dissimilar when compared to all the other age groups (> 94%); whereas, the smallest dissimilarity (70.45%) in microbial community compositions was between the last two sampling points, 125 dph and 193 dph (Table 3). Further analysis of OTU abundance, using LEfSe, identified OTUs that were found to be statistically different in their abundance when comparing 65 dph, 125 dph, and 193 dph, individually back to the origin of the microbiota at 3 dph (S1 Table). Based on this analysis, Clostridia from the genus *Peptostreptococcus* where identified as indicative of the 65 dph catfish microbiota, while *C*. *somerae* was indicative of 125 dph. At 193 dph the catfish microbiota were enriched with specific OTUs from the order Gammaproteobacteria, with LEfSe identifying the families Enterobacteriaceae and Aeromonadales and the genus *Plesiomonas* as indicative of the last time point sampled in this study.

**Table 3:** Similarity percentage analysis (SIMPER) of the most discriminatory bacterial taxa detected in the intestine of channel catfish sampled at various ages (days post hatch; DPH). Prior to the analysis, OTUs were filtered to remove those detected in less than three individual samples. Only those OTUs which accounted for the top 70% of between group dissimilarity are listed.

Group 1 Group 2 (Dissimilarity)	Discriminatory Bacterial Taxa (Order | Genus)	Relative Abundance (%)		Dissimilarity Contribution (%)
		Group 1 Mean	Group 2 Mean	
3DPH 65DPH **(94.48%)**	Alphaproteobacteria|Bradyrhizobium	37	13	21.14
	Bacilli|Streptococcus	0	31	16.33
	Gammaproteobacteria|Plesiomonas	0	27	14.13
	Betaproteobacteria|Comamonadaceae*	20	0	10.56
	Bacilli|Lactobacillus	20	0	10.55
65DPH 125DPH **(87.51%)**	Fusobacteriia|Cetobacterium	15	83	41.56
	Bacilli|Streptococcus	31	0	17.63
	Gammaproteobacteria|Plesiomonas	27	0	15.31
125DPH 193DPH **(70.45)**	Fusobacteriia|Cetobacterium	83	31	39.14
	Gammaproteobacteria|Plesiomonas	0	43	30.31
	Gammaproteobacteria|Enterobacteriaceae*	16	0	11.67
3DPH 125DPH **(99.85%)**	Fusobacteriia|Cetobacterium	0	83	41.42
	Alphaproteobacteria|Bradyrhizobium	37	0	18.75
	Betaproteobacteria|Comamonadaceae*	20	0	10.00
3DPH 193DPH **(99.25%)**	Gammaproteobacteria|Plesiomonas	0	43	21.45
	Alphaproteobacteria|Bradyrhizobium	37	0	18.86
	Fusobacteriia|Cetobacterium	0	31	15.66
	Betaproteobacteria|Comamonadaceae*	20	0	10.06
	Bacilli|Lactobacillus	20	0	10.05
65DPH 193DPH **(80.89%)**	Gammaproteobacteria|Plesiomonas	27	43	26.27
	Fusobacteriia|Cetobacterium	15	31	21.99
	Bacilli|Lactobacillus	31	0	19.07
	Alphaproteobacteria|Bradyrhizobium	13	0	7.75

*—OTU unidentified at the genus level, family level taxonomy is listed

### Environmental Microbiota

**Fig 5:**
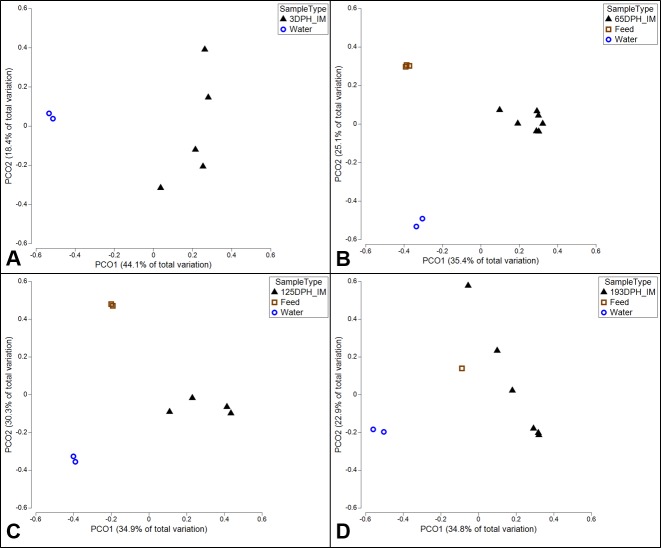
PCoA plots of unweighted UniFrac comparing the intestinal microbiota of channel catfish to that of environmental samples at each time point samples. At all time points, the water microbiota is represented by two replicate samples taken at the end of the trial (193 dph) from the inflowing water supplied to the culture tank. (A) At 3 dph the larval sac-fry had not received any exogenous diets, so environmental comparisons are restricted to the replicate water-supply samples. At 65 dph (B), 125 dph (C), and 193 dph (D) the catfish gut microbiota samples are compared to the replicate water supply samples, as well as the microbiota of all diets unique to that time point.

The environmental microbiota in this study, consisting of six diet samples and two replicate water-supply samples, showed much greater overall microbial diversity as compared to the intestinal samples, with both Chao1 and PD showing significantly greater diversity in the environmental samples at each time point (two-tailed Welch t-test, P ≤ 0.05) (Fig 2 and S2 Table). The most abundant OTU in water, *Crenothrix* (44.9% relative abundance), represented 25.79% of the OTUs detected in the 3 dph sac-fry microbiota, but less than 0.1% in all other fish samples. Conversely, the second most abundant OTU in water (26.5%), Methylophilaceae, was only found to represent 0.2% of the sac-fry microbiota and less than 0.1% of the relative abundance of the microbiota sampled at later dates. In addition, the most abundant microbe detected in the of 3 dph sac-fry, Bradyrhizobium (37%), was present in the water, but at less than 0.1% of the relative abundance. Interestingly, bacteria from the order Lactobacillales, an order from which many commonly used probiotic species are derived, was detected at relatively high levels (41.4%) in the diets fed up to 65 dph, while the diets fed at later points contained less than 5% of Lactobacillales. This may explain the presence of Lactobacillales at relatively high abundance in the 65 dph catfish (19.2%; Fig 4), as well as the fact that OTUs from this order represented less than 0.1% of the relative abundance in the catfish gut after the diets were switched at 125 or 193 dph. Also of interest, *C*. *somerae*, the OTU which was the most abundant microbiota found in the catfish gut at 65, 125, and 193 dph (> 41%) was found to account for only 8% of the relative abundance of OTUs within each of the diets used throughout the study and represents less than 0.01% of the OTUs detected in the water. When comparing the composition of microbial communities which were present in fish to those present in the environment (diets and water) at each time point, using PCoA of unweighted UniFrac, clear separation is apparent (Fig 5). Although Fig 5D may appear to indicate that the 193 dph catfish microbiota was more similar to the microbiota of the diet fed to that time point than that at other time points, an ANOVA showed no differences when comparing the unweighted UniFrac distances between individual gut samples at each time point to the microbiota of either the replicate water-supply samples (mean ± SD; 0.864 ± 0.03) or the diets unique to each time point (0.89 ± 0.034; S3 Table).

## Discussion

The microbiota present in the intestines of animals are suggested to confer an auxiliary genome to their host [52]; yet, environmental and host-associated factors have been shown to influence bacterial membership and act as selective forces on the genetic and metabolic potential of the gut microbiome. Therefore, for the aquaculture industry to promote gains in fish growth and health through microbiota manipulation (pre- and probiotics) we must first gain a better understanding of the structural dynamics which naturally occur in the intestinal microbiota of cultured fish species. In the present study, we have characterized the influence of developmental ontogeny of the host, as well as the concomitant ontogeny of diet, on the microbial communities that inhabit the digestive tract of the commercially valuable channel catfish. As the first temporal study on the gut microbiota of catfish, our results suggest that the host-associated factor of fish age has a significant influence on the ecology of the intestinal microbiota in channel catfish. Therefore, the microbial variability across catfish ontogeny, as discussed below, should be considered when conducting future research on the biology and culture of channel catfish, particularly that related to microbiota manipulations such as pre- and probiotic supplementation.

### Ontogeny of the Catfish Gut Microbiota

The alpha diversity detected in the intestines of juvenile channel catfish in this study was relatively low and consistent across age in both the number of observed OTUs (S2 Fig) and Chao1 species richness estimates (Fig 2A), suggesting somewhat simple microbial communities inhabit the catfish digestive system. Phylogenetic diversity was also low, although a slight yet significant increasing trend in alpha diversity across fish age was detected using this index (Fig 2B). Similar to our findings of relatively low observations of species richness, the number of observed OTUs detected in aquaculture-reared turbot *Scophthalmus maximus* ranged from 7 to 43 OTUs depending on the section of gut being examined [7]. Likewise, Romero et al. [31] described the juvenile coho salmon gut microbiota as rather simple bacterial communities as well, in a study using denaturing gradient gel electrophoresis to explore the microbiome of early life stage coho salmon. Conversely, the only other published research on channel catfish gut microbiota found much higher alpha diversity using 16S sequencing conducted on a combined pool of distal intestinal contents from five individual catfish (observed OTUs = 136) [36]. Although, that study was conducted on a feral pond-raised population of much larger channel catfish (> 500mm in length) of an unknown age [36]. The relatively low alpha diversity detected in the present study may be partially explained by our use of Povidone-iodine treatment at the time of egg collection, an industry standard practice, as well as our rearing of fish in an indoor controlled environment. In addition, the robust OTU filtering applied in this study, including the removal of singletons, non-functional taxa, and OTUs with an abundance below 0.0001%, further reduced alpha diversity as compared to early studies which typically utilized less conservative OTU classification strategies.

The unweighted UniFrac distance based PCoA and nMDS plots (Fig 3) highlight the variability of beta diversity detected in the catfish gut microbiota in this study, both within and between age groups. Further statistical analysis of the beta diversity in this study using PERMANOVA showed that multiple shifts in the gut microbial composition occurred across fish age, while ANOSIM supported these results with the detection of distinguishable separation between specific age groups (Table 2B). Ontogenetic and temporal changes in microbial beta diversity have been detected in other aquaculture fish species as well, such as rainbow trout [33], Atlantic cod [32], and coho salmon [31]. Similar microbiota shifts are commonly detected in other organisms too, including zebrafish [13, 30], humans [53–56], rodents [57, 58], and terrestrial mono-gastric livestock, such as pigs and chickens [59–60]. A common theme among such research is that the newly colonized gut microbiomes of adolescent or early life stage hosts tend to exhibit higher interindividual variability and greater sensitivity to alterations, as compared to the more mature microbiomes of older hosts. Results of the present study are in agreement with this theme, as the microbiota of 3 dph sac-fry larvae showed the greatest interindividual variation, and as a group, the larvae showed the highest dissimilarity when compared to all other time points (Table 3). In addition, the pairwise PERMANOVAs conducted in this study indicate that significant shifts in microbial composition occurred in the catfish gut across the first three time points sampled in this study (P ≤ 0.05; Table 2B). However, pairwise comparisons between the last two time points in this study, 125 and 193 dph, showed no shift in microbial composition (P = 0.0705) and the least amount of separation (ANOSIM R^o^ = 0.190) between all chronological pairwise comparisons (Table 2B). Together, the overall pattern of beta diversity detected in this study may suggest that at early developmental stages dynamic host-microbiota interactions lead to high variability in the catfish gut microbiota, yet as the rate of developmental change slows, the host’s physiology places more consistent regulation upon the microbiota. For example, at the time of hatch, channel catfish immunity is limited to an under-developed non-specific innate immune response, with antibody-mediated humoral immunity not initiated until 21 dph, and the spatial distribution of leukocytes continues to be developmentally dynamic across the first two to six months post hatch [61]. This ongoing maturation of the host immune system, as well as other concurrent morphological and physiological changes occurring in the catfish, could produce dynamic host interactions with the microbiota [25]. These developmental changes, coupled with the nutritional progression from endogenous feeding to consuming prepared diet formulations [33] likely explains the pattern of observed shifts up to 125 dph. The lack of a significant difference in the pairwise PERMANOVA and ANOSIM between the last two time points in this study may suggest that the gut microbiota of these catfish have begun to reach a homeostatic equilibrium at this stage. However, relatively low and uneven sample sizes within this study limit further interpretation at this time, and continued research on the temporal dynamics of the channel catfish intestinal microbiota is needed to determine whether further microbial shifts occur beyond the timeframe of the present study, such as up to market size (0.5–0.7 kg) or sexual maturity (2–3 years post hatch).

### Temporally Differential Taxa

Further evaluation of differentially abundant OTUs across time points can give greater insights into the particular taxa which drove the shifts in beta diversity. One such OTU, *Bradyrhizobium*, was identified by SIMPER as the most characteristic OTU of the 3 dph larval samples (Table 3), as it was the most abundant OTU in the larval sac-fry (37%; Rhizobiales in Fig 4). However, *Bradyrhizobium* represented only 1.3% of the OTUs detected in the 65 dph catfish microbiota and less than 0.1% in all other samples, both intestinal and environmental. Interestingly, this OTU represents a genus of bacteria that is typically found in soil as a symbiont to plants by supplying nitrogen-fixation [62]. Although recently, *Bradyrhizobium enterica* was discovered in the human gut and was suggested as the etiology behind human intestinal cord-colitis syndrome [63], suggesting these bacteria may be capable of membership in a vertebrate gut microbiome. In addition, bacteria from the same family (Bradyrhizobiaceae) were detected in the microbiome of larval Atlantic cod, albeit at very low abundance (0.1%) [32]. In opposition, Laurence et al. [64] rather convincingly showed *Bradyrhizobium* to be a common contaminant of sequencing datasets, with contamination particularly affecting low biomass samples [65]. This may explain the much higher abundance of *Bradyrhizobium* in the relatively low biomass 3 dph larval samples; however, no *Bradyrhizobium* was detected in the negative control in this study (S3 Fig). Therefore, further studies are required to fully discern whether this OTU is a true inhabitant of the channel catfish digestive tract or possibly a laboratory contaminant.

Excluding the 3 dph sac-fry larvae, the dominant OTU in all other catfish intestinal samples in this study was *Cetobacterium somerae*, which represented the only taxa from the phylum Fusobacteria detected in the gut samples (Fig 4). This OTU is a microaerotolerant anaerobe that is capable of producing vitamin B-12 and antimicrobial metabolites [66], suggesting the host may derive physiological benefits from this microorganism. In agreement with our findings, the one previous study conducted on the intestinal microbiota of channel catfish, found *C*. *somerae* to represent 94.02% of the bacterial abundance detected in a pooled sample of intestinal contents from five catfish [36]. These findings suggest that *C*. *somerae* is a highly abundant commensal core microbe within the intestine of channel catfish. Interestingly, this species of Fusobacteria and a few close phylogenetic relatives were detected and discussed in great detail in a comprehensive survey of core microbes in the zebrafish gut as well [67]. In this study, *Plesiomonas shigelloides* were detected at high levels at 65 dph (30.8%) as well as 193 dph (26.1%), but only at very low levels at 3 and 125 dph (≥ 0.1%). *P*. *shigelloides* from the Enterobacteriaceae family of Proteobacteria is commonly detected in freshwater fish and aquatic environments, and strains isolated from the fish gut have been shown to possess antimicrobial abilities [68]. LEfSe analysis indicated that OTUs within the families Enterobacteriaceae and Aeromonadaceae were significantly more abundant in the catfish gut at 193 dph than at any other time point (S1 Table; Fig 4). At the species level, bacteria within both of these families have been shown to serve as symbiotic and commensal microbiota [66], while others are potent fish pathogens [68–70]. Unfortunately, these OTUs could not be identified further than the family level, so it is impossible to determine the function of those OTUs within this study. *Streptococcus*, *P*. *shigelloides*, Enterobacteriaceae, and Aeromonadales are all OTUs that were detected in the gut of channel catfish after 3 dph, and all exhibited differential abundance across fish age. In addition, these OTUs have all been implicated in pathogenic outbreaks in freshwater fish species, while also being detected in commensal relationships with their fish host, as all of these OTUs also possess antimicrobial properties [68–72]. Despite the presence of these known potential pathogens, the fish population sampled within this study exhibited no mortality or signs of disease throughout the experiment, and these potentially pathogenic microbes were never the most dominant OTUs in the catfish gut. The temporal variance and noted potential for virulence of these OTUs may suggest that they function as opportunistic pathogens in the catfish gut, requiring some major breach in host homeostasis before becoming virulent.

### Influence of Environmental Microbiota

The aquatic environment in which fish live is perfectly suited to the colonization and growth of microorganisms, potentially leading to higher levels of interaction between environmental and host associated microbiota as compared to the environments of terrestrial animals [29]. Therefore the environmental microbiota may be of even greater consequence to fish than that of a terrestrial species [73]. In this study, *Crenothrix* was the most abundant OTU found in the water-supply samples and represented just over 25% of the OTUs detected in the larval microbiota at 3 dph, while representing less than 0.1% in all later gut samples. This suggest the water microbiota had a stronger influence on the gut at early life stages, while the fish were still feeding endogenously. Although, it must be noted that the replicate water-supply samples used to represent the water microbiota in this study were only available from a single sampling period (193 dph), therefore temporal variation in water microbiota could not be assessed.

In the present study, diet formulation was slightly changed after each sampling period to meet the changing nutritional requirements of the developing catfish, therefore the changes in microbiota composition detected over time are also influenced by dietary changes. However, it is difficult to separate the influence of ontogeny from changes in diet, as most aquacultured fish species have significant changes in dietary requirements across early development [74]. Thus, dietary changes are intrinsic to ontogenetic studies. Additionally, microbiota comparisons between the catfish gut and the administered diets offer greater insight into the influence of the diet-derived microbes on the intestinal microbiota of the catfish. For instance, bacteria from the genus *Streptococcus* accounted for 19.2% of the relative abundance of the catfish gut microbiome at 65 dph, while accounting for less than 0.1% in all other gut samples. The microbiota of the diets fed at 65 dph also showed a high abundance of *Streptococcus* (12.4%), yet this OTU represented only 0.1% of the administered diet microbiota at 125 dph, and 5% at 193 dph. This somewhat parallel pattern in *Streptococcus* abundance between the catfish intestinal microbiota and the microbiota of the associated diet samples suggests that diet may have introduced this OTU to the gut microbiota, which could be useful when considering effective routes of probiotic supplementation. Similarly, Smith and coworkers showed that differences in diet-derived microbes associated with the prey items of geographically separate populations of three-spine stickleback partially explained the differences detected in gut microbiota across the various populations [75]. Although when employing sequence based analysis, discerning if OTUs were detected as transient diet-derived microbial DNA artifacts or as viable functioning members of the gut microbiota is difficult and would require further studies. Therefore, it is likely that the detection of *Streptococcus* in the gut microbiota at 65 dph was an artifact of transient diet-derived microbial DNA, given that the OTU did not persist at later sampling points (125 and 193 dph).

On aggregate, the intestinal microbial communities sampled in this study were maintained separate from the environmental microbiota (water-supply and administered diets). Significant differences in alpha diversity were detected between fish samples and environmental samples at each time point, with the environmental samples showing much greater alpha diversity (Fig 2; S3 Table). Furthermore, comparisons of beta diversity UniFrac distances showed consistent separation between the environmental and intestinal microbiota at each time point (Fig 5), and UniFrac distances between gut samples and environmental samples did not change over time (P > 0.05; S3 Table). In addition, many of the microbes which showed high abundance in gut samples were detected at relatively low levels in the environmental microbiomes in this study. This suggests the physiological and morphological features associated with the catfish digestive tract create a unique ecological niche, maintained separate from the aquatic and diet derived microbes (S3 Table). This finding is corroborated by microbiota ontogeny studies conducted on other fish species [23, 30, 32] that show clear separation between the fish gut and the environment (diet and water).

## Conclusion

In summary, the present study represents the first ontogenetic characterization of the intestinal microbiota of the commercially and scientifically important fish species, channel catfish. When controlling for environmental and genetic variability, shifting microbial communities were detected in a single population of channel catfish across fish age; however, the required changes in dietary formulations across development is likely to have influenced microbiota composition as well. Despite these shifts of the gut microbiota, shared bacteria were also identified across fish age, with *Cetobacterium somerae* found to be a dominant bacterial species in the intestinal tract of catfish after the larval stage, irrelevant of age. And while pathogenic bacterial taxa were detected at each time point, fish exhibited no signs of disease suggesting those pathogenic bacteria may be commensal microorganisms that are opportunistically pathogenic only when the host’s physiology deviates from homeostasis. In addition, we showed that the intestinal microbiota of channel catfish are distinct from the surrounding environmental microbiota. However, the environment (diet and water-supply) likely serves as an inoculum to the fish gut, as there were shared taxa between intestinal and environmental samples as well. The results of this study have implications on the applied management of catfish aquaculture and currently represent the only temporal characterization of the gut microbiota of channel catfish. Our results highlight the variability in gut microbiota between individuals and age classes as a potential source of variation when conducting other research on the biology and culture of these commercially valuable fish, as well as serve as a reference for future research on pre- and probiotic supplementation in catfish. Furthermore, these results help increase our basic understanding of temporal variation in the microbial composition dynamics in the gastrointestinal tract of teleost fish.

## Supporting Information

S1 Fig Primer sequences used for construction of 16S rRNA V4 gene amplicon sequencing library and diagram of final amplicon construct. (PDF)

S2 Fig Rarefaction curves of observed OTUs detected in the intestinal microbiota of channel catfish. (PDF)

S3 Fig Comparison of the OTUs detected in a negative control water sample and the replicate water-supply samples. The OTUs detected in the negative control sample are considered contaminate sequences within this study. The absolute count abundances of each OTU detected in the negative control sample are listed below the bar plot.
(PDF)

S1 Table Least Discriminate Effect Size (LEfSe) analysis of the abundance of bacterial taxa detected in the intestinal tract of channel catfish at 3, 65, 125, and 193 days post hatch (DPH). LEfSe analysis conducted using the all-against-one model (Kruskal-Wallis test p ≤ 0.05; log 10 LDA score threshold of 2.0).
(XLSX)

S2 Table Two-tailed Welch t-tests conducted on the alpha diversity (Chao1 and Phylogenetic Distance) detected in channel catfish gut microbiota and the environmental microbiota at each time point sampled: 3, 65, 125, and 193 days post hatch (DPH). (PDF)

S3 Table ANOVAs conducted on the average unweighted UniFrac distances between the gut microbiota of channel catfish and the environmental microbiota associated with the water-supply and the diets administered at each time point: 3, 65, 125, and 193 days post hatch (DPH). At 3 dph fish had received no diets, therefore 3 dph was not included in comparisons to diet microbiota.
(PDF)
