# Supplementary material for: Ontogenetic Characterization of the Intestinal Microbiota of Channel Catfish through 16S rRNA Gene Sequencing Reveals Insights on Temporal Shifts and the Influence of Environmental Microbes
Source: PLoS One. 2016 Nov 15;11(11):e0166379. doi: 10.1371/journal.pone.0166379 (PMC5113000; doi:10.1371/journal.pone.0166379)

Region	Primer Name	Primer Sequence
16S rRNA V4	515F	5'-GTGCCAGCMGCCGCGGTAA
	806R	5'-GGACTACHVHHHTWTCTAAT
Fluidigm Specific Primer Pads	CS1	5'-ACACTGACGACATGGTTCTACA
	CS2	5'-TACGGTAGCAGAGACTTGGTCT
Indexing Barcode	Index	XXXXXXXXXX (Unique to each sample)
Illumina Specific Sequencing Primers	i5	5'-AATGATACGGCGACCACCGAGATCT
	i7	5'-CAAGCAGAAGACGGCATACGAGAT

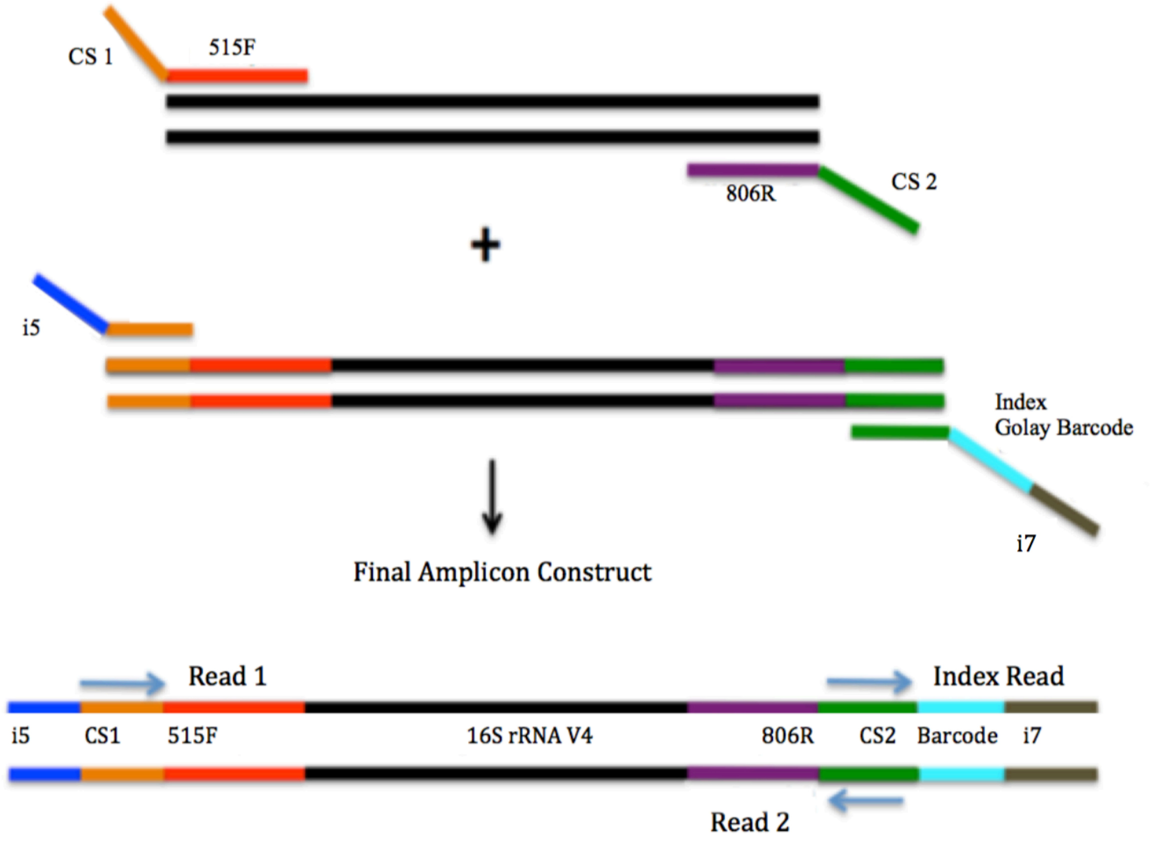

Supplement: S1 Fig — (PDF) [file pone.0166379.s001.pdf]
