# Supplementary material for: Ontogenetic Characterization of the Intestinal Microbiota of Channel Catfish through 16S rRNA Gene Sequencing Reveals Insights on Temporal Shifts and the Influence of Environmental Microbes
Source: PLoS One. 2016 Nov 15;11(11):e0166379. doi: 10.1371/journal.pone.0166379 (PMC5113000; doi:10.1371/journal.pone.0166379)
Supplement: S2 Fig — (PDF) [file pone.0166379.s002.pdf]

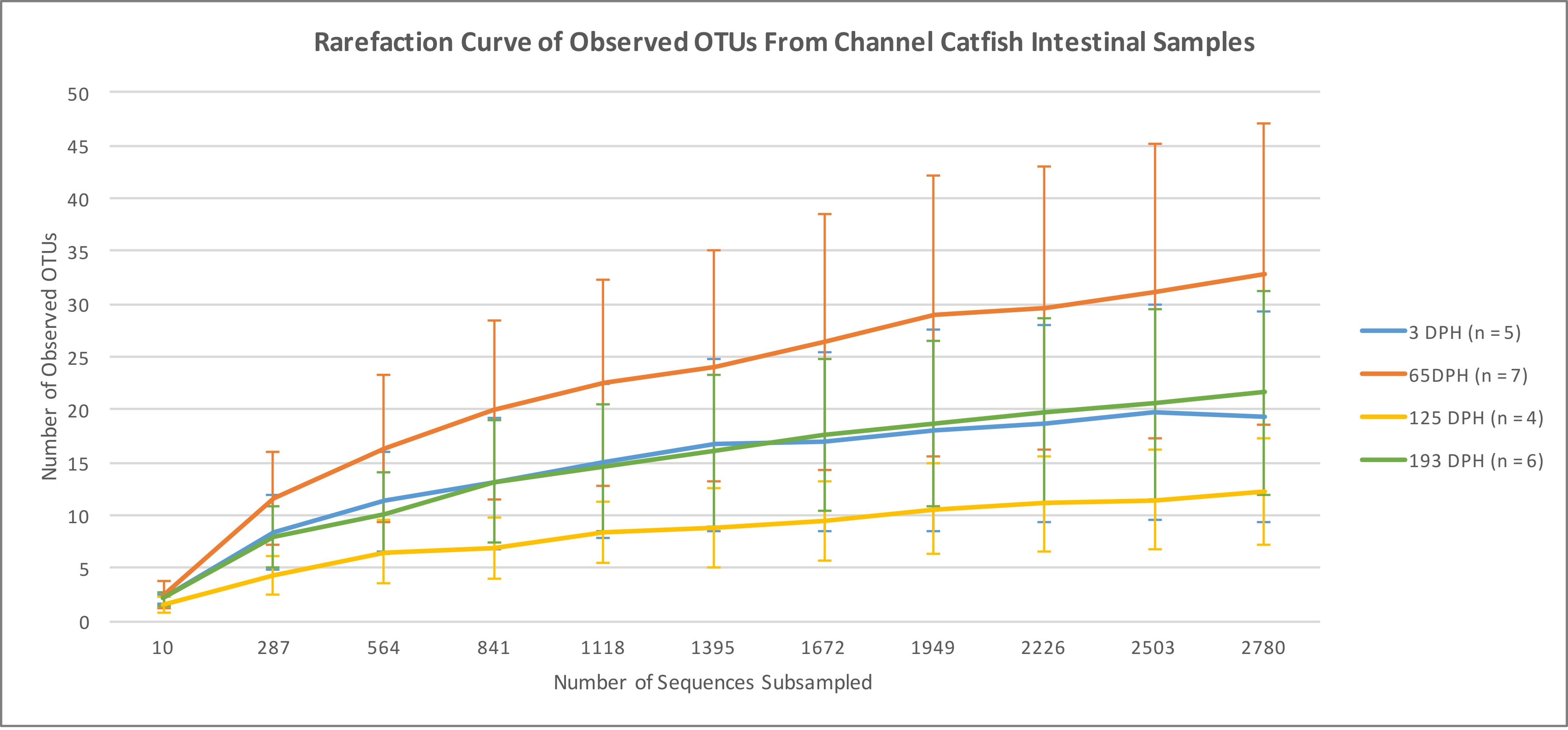
Rarefaction Curve of Observed OTUs From Channel Catfish Intestinal Samples
Number of Observed OTUs
Number of Sequences Subsampled
0
5
10
15
20
25
30
35
40
45
50
10
287
564
841
1118
1395
1672
1949
2226
2503
2780
3 DPH (n = 5)
65DPH (n = 7)
125 DPH (n = 4)
193 DPH (n = 6)

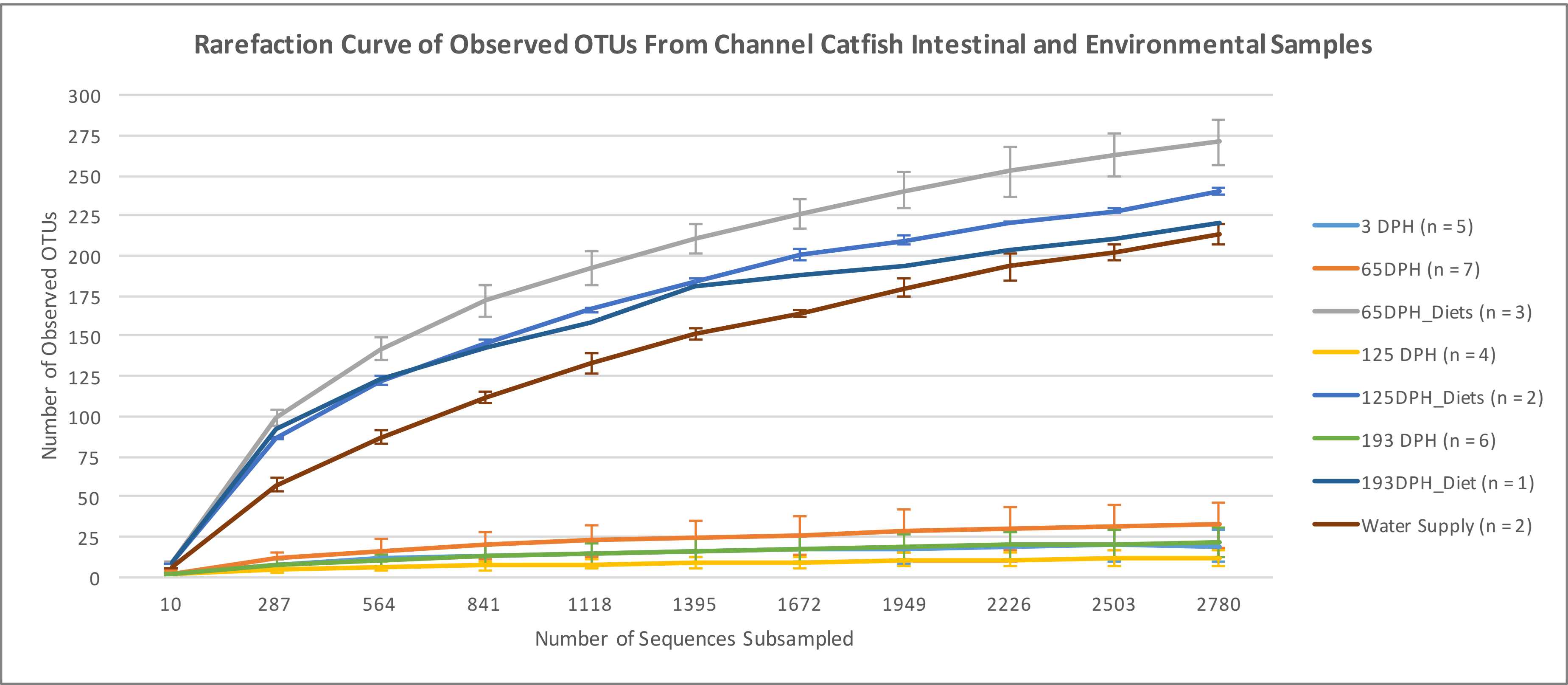
Rarefaction Curve of Observed OTUs From Channel Catfish Intestinal and Environmental Samples
Number of Observed OTUs
Number of Sequences Subsampled
0
25
50
75
100
125
150
175
200
225
250
275
300
10
287
564
841
1118
1395
1672
1949
2226
2503
2780
3 DPH (n = 5)
65DPH (n = 7)
65DPH_Diets (n = 3)
125 DPH (n = 4)
125DPH_Diets (n = 2)
193 DPH (n = 6)
193DPH_Diet (n = 1)
Water Supply (n = 2)
