# Supplementary material for: Ontogenetic Characterization of the Intestinal Microbiota of Channel Catfish through 16S rRNA Gene Sequencing Reveals Insights on Temporal Shifts and the Influence of Environmental Microbes
Source: PLoS One. 2016 Nov 15;11(11):e0166379. doi: 10.1371/journal.pone.0166379 (PMC5113000; doi:10.1371/journal.pone.0166379)
Supplement: S3 Fig — The OTUs detected in the negative control sample are considered contaminate sequences within this study. The absolute count abundances of each OTU detected in the negative control sample are listed below the bar plot. (PDF) [file pone.0166379.s003.pdf]

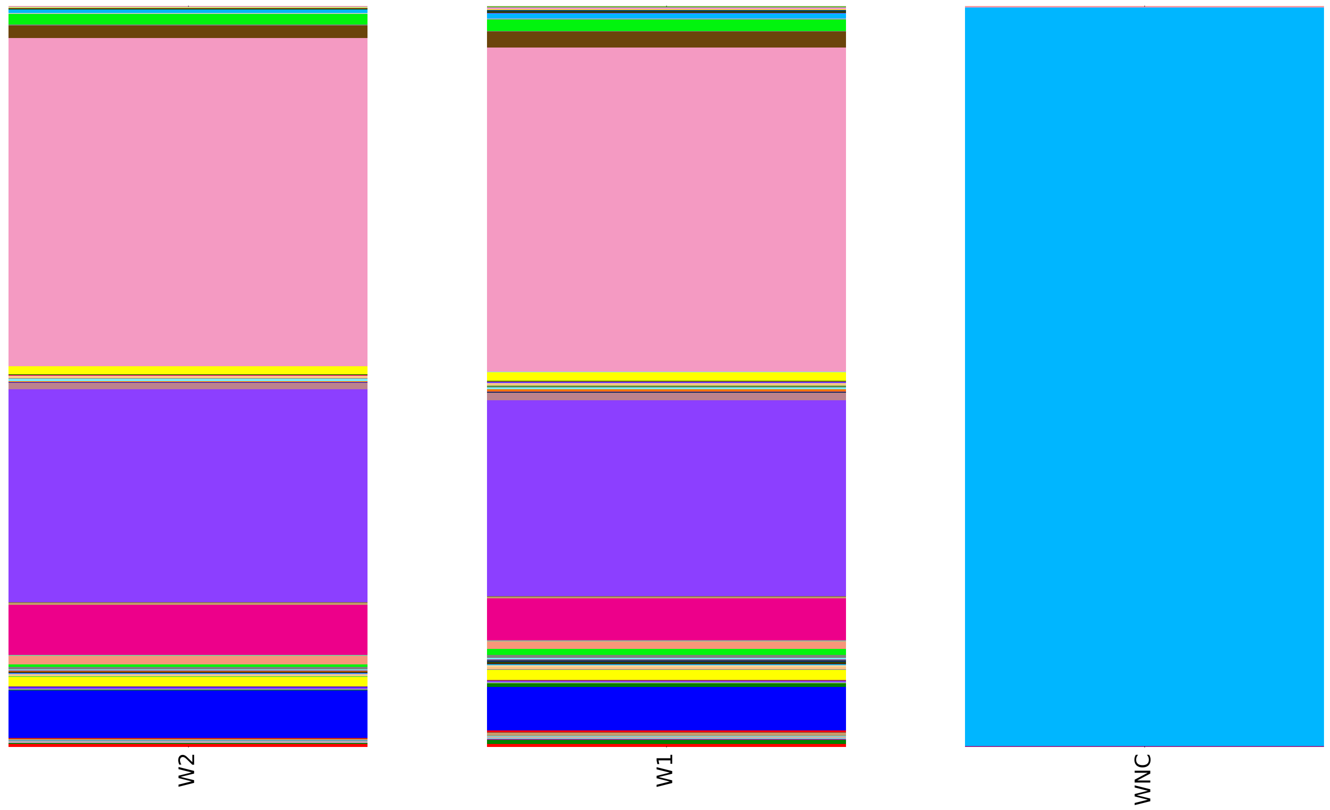


| OTU Taxonomy | Replicate Water Supply | | Neg. Control |
|---|---|---|---|
| | W1 | W2 | WNC |
| Fusobacteria\|Fusobacteriia\|Fusobacteriales\|Fusobacteriaceae\|Cetobacterium | 45 | 65 | 2 |
| Proteobacteria\|Gammaproteobacteria\|Methylococcales\|Crenotrichaceae\|Crenothrix | 66,227 | 74,921 | 2 |
| Proteobacteria\|Alphaproteobacteria\|Rhizobiales\|Methylobacteriaceae\|Methylobacterium | 16 | 1 | 1,217 |
| | | | |
| Total number of OTUs | 151,315 | 169,262 | 1,221 |
