# Supplementary material for: Ontogenetic Characterization of the Intestinal Microbiota of Channel Catfish through 16S rRNA Gene Sequencing Reveals Insights on Temporal Shifts and the Influence of Environmental Microbes
Source: PLoS One. 2016 Nov 15;11(11):e0166379. doi: 10.1371/journal.pone.0166379 (PMC5113000; doi:10.1371/journal.pone.0166379)
Supplement: S2 Table — (PDF) [file pone.0166379.s005.pdf]

Alpha Diversity Index	Groups Compared		Group 1 Mean	Group 2 Mean	Test Statistic (t)	df	P value
**Chao1 Richness Estimator**	3 DPH Sac-fry Larvae	3 DPH Environment	19.90	250.21	-28.50	2.10	0.0009
	65 DPH Intestine	65 DPH Environment	62.17	307.69	-8.64	6.94	0.0001
	125 DPH Intestine	125 DPH Environment	44.00	282.41	-9.28	5.92	0.0001
	193 DPH Intestine	193 DPH Environment	49.06	255.34	-15.36	6.88	0.0001
**Phylogenetic Diversity**	3 DPH Sac-fry Larvae	3 DPH Environment	2.17	9.88	-10.67	1.42	0.0252
	65 DPH Intestine	65 DPH Environment	3.68	13.73	-6.04	4.28	0.0030
	125 DPH Intestine	125 DPH Environment	2.76	13.16	-5.33	3.24	0.0106
	193 DPH Intestine	193 DPH Environment	3.30	11.30	-5.41	2.05	0.0307
