# Supplementary material for: Ontogenetic Characterization of the Intestinal Microbiota of Channel Catfish through 16S rRNA Gene Sequencing Reveals Insights on Temporal Shifts and the Influence of Environmental Microbes
Source: PLoS One. 2016 Nov 15;11(11):e0166379. doi: 10.1371/journal.pone.0166379 (PMC5113000; doi:10.1371/journal.pone.0166379)
Supplement: S3 Table — At 3 dph fish had received no diets, therefore 3 dph was not included in comparisons to diet microbiota. (PDF) [file pone.0166379.s006.pdf]

Intestinal Microbiota vs Water Supply Microbiota (Unweighted UniFrac Distances)

Mean Distances from Water Supply

	3DPH	65DPH	125DPH	193DPH
Fish 1	0.8187	0.8908	0.9153	0.8936
Fish 2	0.8810	0.9033	0.8566	0.8879
Fish 3	0.9113	0.9055	0.8049	0.8848
Fish 4	0.9116	0.8825	0.9249	0.9115
Fish 5	0.9175	0.8596		0.9581
Fish 6		0.8824		0.8894
Fish 7		0.8927		

Average Distance	0.8902
Standard Deviation	0.0336

Summary Statistics

Groups	*Count*	*Average*	*Variance*
3DPH	5	0.8880	0.0017
65DPH	7	0.8881	0.0002
125DPH	4	0.8755	0.0031
193DPH	6	0.9042	0.0008

ANOVA

Source of Variation	*df*	*F*	*P-value*
Between Groups	3	0.5854	0.6323
Within Groups	18		
Total	21		

Intestinal Microbiota vs Diet Microbiota (Unweighted UniFrac Distances)

Mean Distances from Administered Diets

	65 DPH	125 DPH	193 DPH
Fish 1	0.8082	0.9216	0.8592
Fish 2	0.8573	0.8378	0.8376
Fish 3	0.8381	0.8572	0.8546
Fish 4	0.8833	0.9136	0.9062
Fish 5	0.8298		0.9279
Fish 6	0.8595		0.8076
Fish 7	0.8916		

Average Distance	0.8642
Standard Deviation	0.0375

Summary Statistics

Groups	*Count*	*Average*	*Variance*
65 DPH	7	0.8525	0.0009
125 DPH	4	0.8826	0.0017
193 DPH	6	0.8655	0.0020

ANOVA

Source of Variation	*df*	*F*	*P-value*
Between Groups	2	0.8008	0.4685
Within Groups	14		
Total	16		
